# TNF-α Induces Mitophagy in Rheumatoid Arthritis Synovial Fibroblasts, and Mitophagy Inhibition Alleviates Synovitis in Collagen Antibody-Induced Arthritis

**DOI:** 10.3390/ijms23105650

**Published:** 2022-05-18

**Authors:** Ji-Hee Nam, Jun-Ho Lee, Hyun-Ji Choi, So-Yeon Choi, Kyung-Eun Noh, Nam-Chul Jung, Jie-Young Song, Jinjung Choi, Han Geuk Seo, Sang Youn Jung, Dae-Seog Lim

**Affiliations:** 1 Department of Biotechnology, CHA University, 335 Pangyo-ro, Bundang-gu, Seongnam 13488, Korea; wl08gml03@naver.com (J.-H.N.); hjc1991@naver.com (H.-J.C.); nge6@naver.com (K.-E.N.); 2 Pharos Vaccine Inc., 14 Galmachiro, 288 Bun-gil, Jungwon-gu, Seongnam 13201, Korea; jhlee@pharosvaccine.com (J.-H.L.); csy5900@naver.com (S.-Y.C.); ncjung@pharosvaccine.com (N.-C.J.); 3 Department of Radiation Cancer Sciences, Korea Institute of Radiological and Medical Sciences, 75 Nowon-ro, Nowon-gu, Seoul 01812, Korea; immu@kcch.re.kr; 4 CHA Bundang Medical Center, Department of Internal Medicine, Division of Rheumatology, CHA University School of Medicine, Seongnam 13496, Korea; jinjung@chamc.co.kr; 5 Department of Food Science and Biotechnology of Animal Products, Sanghuh College of Life Sciences, Konkuk University, 120 Neungdong-ro, Gwangjin-gu, Seoul 05029, Korea; hgseo@konkuk.ac.kr

**Keywords:** mitophagy, PINK1, rheumatoid arthritis, synovial fibroblast, TNF-α

## Abstract

Mitophagy is a selective form of autophagy that removes damaged mitochondria. Increasing evidence indicates that dysregulated mitophagy is implicated in numerous autoimmune diseases, but the role of mitophagy in rheumatoid arthritis (RA) has not yet been reported. The aim of the present study was to determine the roles of mitophagy in patient-derived RA synovial fibroblasts (RASFs) and in the collagen antibody-induced arthritis mouse model. We measured the mitophagy marker PTEN-induced putative kinase 1 (PINK1) in RASFs treated with tumor necrosis factor-α (TNF-α) using Western blotting and immunofluorescence. Arthritis was induced in *PINK1*^−/−^ mice by intraperitoneal injection of an anti-type II collagen antibody cocktail and lipopolysaccharide. RA severity was assessed by histopathology. PINK1 expression and damaged mitochondria increased in TNF-α treated RASFs via increased intracellular levels of reactive oxygen species. PINK1 knockdown RASFs decreased cellular migration and invasion functions. In addition, *PINK1*^−/−^ mice with arthritis exhibited markedly reduced swelling and inflammation relative to wild-type mice with arthritis. Taken together, these findings suggest that regulation of PINK1 expression in RA could represent a potential therapeutic and diagnostic target for RA.

## 1. Introduction

Rheumatoid arthritis (RA) is a common autoimmune disease affecting 1% of the global population. Active RA involves chronic inflammation and joint destruction driven by proliferating RA synovial fibroblasts (RASFs) and leukocyte trafficking in the synovium [1]. Activated RASFs express adhesion molecules such as integrins of the β1 superfamily. Adhesion molecule upregulation mediates attachment of RASFs to fibronectin-rich cartilage sites [2]. The matrix degradation enzymes (MMPs) and cathepsins are expressed by activated RASFs and contribute to cartilage and bone destruction. As RASFs mediate most joint destruction pathways in RA, molecular insights into these cells could identify important targets for novel therapeutic approaches that inhibit cartilage and bone destruction in RA [3,4,5]. When synovitis occurs, leukocytes accumulate in the synovial compartment. Activated macrophages, monocytes, and neutrophils release cytokines, including tumor necrosis factor-α (TNF-α), interleukin-1, and Interleukin-6 [6,7]. TNF-α induce autophagy by increasing the generation of reactive oxygen species (ROS), but RASFs are relatively resistant to TNF-α-induced apoptosis [8,9].

Autophagy affects cell survival by preserving cellular bioenergetics and clearing dysfunctional organelles, and misfolded proteins [10]. During autophagy, a cytoplasmic form of LC3 (LC3-I) is converted to the LC3-phosphatidylethanolamine conjugate (LC3-II), which is recruited to the autophagosomal membrane. Autophagosomes fuse with lysosomes to form autolysosomes, and components within autohagosomes are degraded by lysosomal hydrolases [11] A selective form of autophagy, termed mitophagy, is a major mechanism for the removal of damaged mitochondria in the mitochondrial quality control process. One of the primary mitophagy pathways is ubiquitin E3 ligase Parkin/PTEN-induced putative kinase 1 (PINK1)-mediated mitophagy [12]. PINK1 levels are low in healthy mitochondria, as under normal conditions, PINK1 is rapidly degraded by presenilin-associated rhomboid-like (PARL) protease at the inner mitochondrial membrane that is activated by the SPY complex (SLP2-PARL-YME1L). In damaged mitochondria that lose mitochondrial membrane potential, PINK1 is accumulated on the outer mitochondrial membrane, stimulating the recruitment of Parkin to mitochondria [13]. PINK1-Parkin-dependent signaling induces ubiquitylation cycles on the outer mitochondrial membrane, which results in the formation of the autophagosome and clearance of damaged mitochondria [14]. Parkin E3-ligase activity is regulated by the enzymatic activity of PINK1 [15,16]. PINK1 expression affects human pathology through diverse pathways. Recent reports have suggested that PINK1 is down regulated during the pro-fibrotic response, increasing susceptibility to lung fibrosis [17,18]. Thus, mitochondrial dysfunction and mitochondrial fusion that produces ROS and induces apoptosis are downstream of these effects [19]. ROS generated by disrupted electron transport in damaged mitochondria contribute to Parkinson’s disease by damaging neurons [20]. Mutations in genes involved in mitochondrial metabolism are linked to Parkinson’s disease, including genes such as *PINK1*, *PRKN*, *SNCA*, and *PARK7* [21,22]. Loss-of-function mutations in the *PINK1* gene, which regulates mitophagy, are linked to familial Parkinson’s disease [23,24]. In these patients, due to disrupted PINK1 function, mitochondrial quality control is compromised, and accumulation of damaged mitochondria leads to oxidative stress, which inhibits dopamine secretion and neuronal synaptic transmission, resulting in Parkinson’s disease [25]. Once PINK1 loss-of-function compromises mitophagy, apoptosis is induced and the functions of brain [22] and lung tissues are not maintained properly. PINK1 is thus expected to play an important role in maintaining cell survival under cellular stress.

Although the roles of mitophagy in pathologies of the nervous system and lungs are established, the role of mitophagy in autoimmune diseases such as RA remains incompletely understood. In the present study, when synovial fibroblasts from patients with rheumatoid arthritis were treated with TNF-α, we investigated whether mitophagy was induced in RASFs. Using PINK1 knockdown method assay in RASFs, we demonstrated that mitophagy contributes to cell migration and invasion. Furthermore, we demonstrated the preclinical relevance of PINK-mediated mitophagy using the induction of arthritis in *Pink1^−/−^* mice.

## 2. Results

### 2.1. Measurement of PINK1 Expression in Synovial Tissues of Patients with Osteoarthritis (OA) and RA

**Figure 1:**
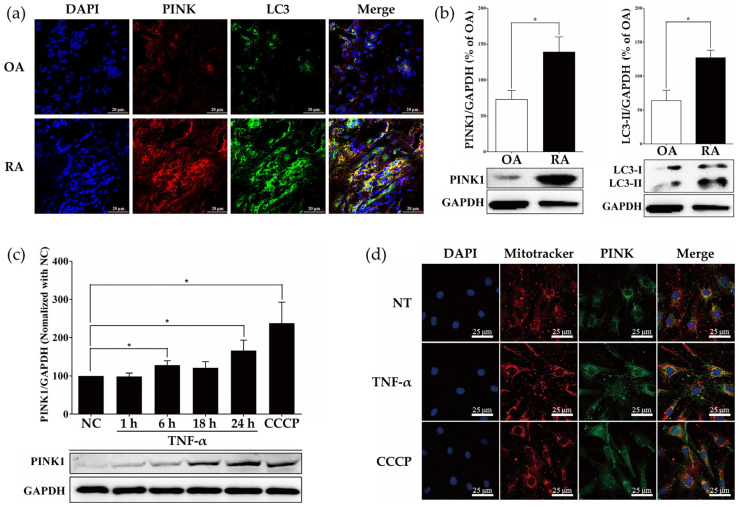
PINK1 is highly expressed in synovial tissues of rheumatoid arthritis patients and TNF-α-treated RASFs. (**a**) Immunofluorescence staining for PINK1 (red), LC3B (green), and nuclei (blue) in synovial membranes of OA and RA patients. Scale bar: 20 μm. Images are representative of five images/sample; *n* = 3 samples/disease group. (**b**) Tissue Western blots for PINK1 and LC3B-II in human synovial membranes from OA (*n* = 3) and RA (*n* = 3) patients. The bar graph shows quantification by densitometry of LC3-II, and PINK1 in synovial membranes from RA and OA patients, presented as means ± SEM (*n* = 3 independent patient synovial membranes). * *p* < 0.05. (**c**) TNF-α (10 ng/mL) induced PINK1 upregulation in a time-dependent manner. RASFs were treated with CCCP (10 μM) for 24 h. PINK1 protein was detected by immunoblotting. The bar graph shows the quantification of PINK1 expression by densitometry in RASFs, expressed as means ± SEM (*n* = 5 independent RASF preparations/group). * *p* < 0.05. (**d**) Immunofluorescence staining for PINK1 (green), mitochondria (red), and nuclei (blue) in RASFs treated with TNF-α for 24 h. Scale bar: 25 μm. Image (original magnification, ×100) is representative of five images/slide, *n* = 3 slides/treatment group.

To investigate mitophagy occurring on the synovial membrane, we performed PINK1 and LC3 staining, using LC3 as a marker of autophagy, in synovial membrane tissues of RA patients and OA patients by immunofluorescence analysis. PINK1 and LC3 expressions were increased in synovial membranes of the RA patients more than in OA patients (Figure 1a). To quantitatively determine the expression of PINK1 and LC3 in the synovial tissue of each patient, tissue protein was extracted and analyzed by Western blot. Protein levels of PINK1 and LC3-II were significantly increased in the tissues of RA patients (Figure 1b). Given the central role of TNF-α in RA, we determined if TNF-α stimulation of RASFs would affect PINK1 protein level. TNF-α induced PINK1 accumulation at 24 h (Figure 1c). RASFs were stimulated with 10 ng/mL TNF-α for 24 h and mitochondrial co-localization with PINK1 was analyzed by immunofluorescence. Although there was less accumulation of PINK1 on mitochondria in TNF-α treated cells compared with carbonyl cyanide m-chlorophenylhydrazone (CCCP), TNF-α induced more accumulation of PINK1 on mitochondria relative to control (Figure 1d). Taken together, these findings suggested that mitophagy was actively induced in the synovial membrane of rheumatoid joints with active inflammation.

### 2.2. ROS Induced by TNF-α Regulate Expression of PINK1

**Figure 2:**
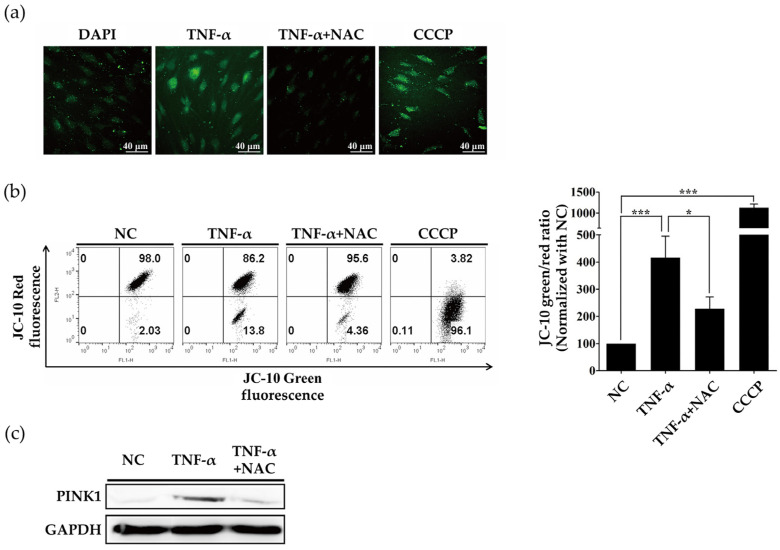
TNF-α stimulation increases ROS levels to induce PINK1 accumulation in RASFs. (**a**) After pretreatment with NAC (5 mM) for 1 h, RASFs were stimulated with TNF-α (10 ng/mL) or CCCP (10 μM) for 24 h. Cells were stained with CM-DCFDA for 30 min. Immunofluorescence revealed that TNF-α induced ROS formation and NAC quenched ROS. Data are representative of at least three independent experiments. (**b**) Cells were stained with JC-10 for 30 min and subjected to flow cytometry. Data are representative of five independent RASF preparations. * *p* < 0.05; *** *p* < 0.001. (**c**) Western blot for PINK1 in RASFs stimulated with TNF-α (10 ng/mL) for 24 h + NAC (5 mM), revealing increased PINK1 levels with TNF-α stimulation and decreased PINK1 levels in TNF-α + NAC co-treated cells.

Mitophagy is a cellular response associated with mitochondrial dysfunction. TNF-α could induce mitochondrial dysfunction, inducing excessive ROS production. To determine the role of ROS in the initiation of mitophagy, we used the ROS scavenger N-acetylcysteine (NAC). ROS increased in response to TNF-α, and NAC effectively removed ROS, despite the presence of TNF-α (Figure 2a). To assess the mitochondrial function, the mitochondrial membrane potential was evaluated using a JC-10 assay. The ratio of red to green fluorescence revealed that control cells exuded red fluorescence, reflecting healthy mitochondria. Treatment of RASFs with TNF-α decreased red fluorescence, indicating decreased mitochondrial membrane potential. However, red fluorescence increased in NAC-treated cells, indicating that quenching ROS allowed the recovery of mitochondrial membrane potential under TNF-α stress (Figure 2b). We also measured PINK1 expression in TNF-α, and NAC-treated RASFs. Co-treatment with NAC abrogated TNF-α induced PINK1 accumulation (Figure 2c).

### 2.3. PINK1 Inhibition Decreased RASF Migration and Invasion

**Figure 3:**
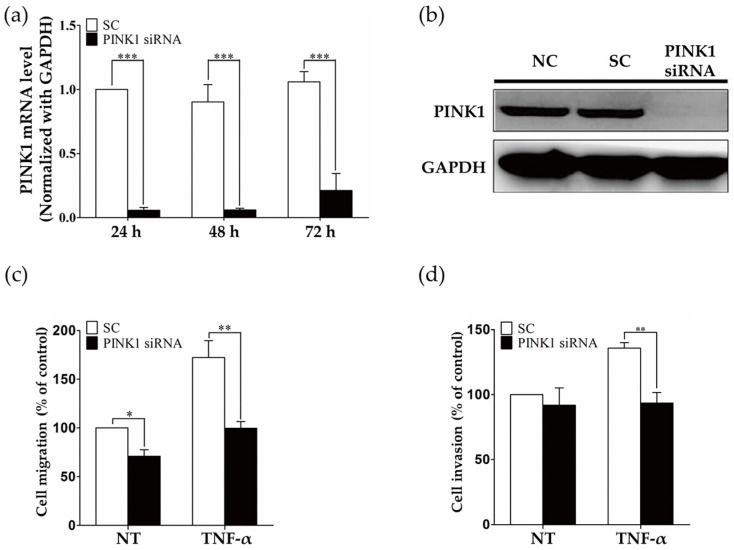
*PINK1* ablation impairs RASF migration and invasion. (**a**) Real-time PCR measurement of *PINK1* mRNA demonstrated that siRNA knockdown of *PINK1* successfully decreased *PINK1* mRNA levels. In total, 10 nM PINK1 siRNA was transfected into RASFs for 24 h, 48 h, and 72 h. Data are expressed as means ± SEM for three independent experiments. ** *p* < 0.01; *** *p* < 0.001. (**b**) Western blot quantification of PINK1 protein demonstrated that PINK1 siRNA knockdown decreased PINK1 protein levels. In total, 10 nM PINK1 siRNA was transfected into RASFs for 24 h. Data are representative of at least three independent experiments. (**c**,**d**) Cell migration and invasion assay in *PINK1* knockdown RASFs. RASFs were transfected with scramble (sc) or PINK1 siRNA and stimulated with TNF-α (10 ng/mL). Data are expressed as means ± SEM for three independent experiments. * *p* < 0.05 *** *p* < 0.001.

Additionally, to provide insight into the function of the PINK1 in RASF migration and invasion of arthritic joints, the *PINK1* gene was silenced by PINK-targeting siRNA, as verified by decreased mRNA and protein levels of PINK1 (Figure 3a,b). Subsequently, we tested migratory and invasive functions. PINK1 knockdown in RASFs significantly decreased migration and invasion under TNF-α conditions (Figure 3c,d). Taken together, these findings suggested that mitophagy was dependent on intracellular ROS generation, and that RASF migration and invasion were decreased when mitophagy was inhibited.

### 2.4. PINK1 Deficiency Ameliorates Collagen Antibody-Induced Arthritis

**Figure 4:**
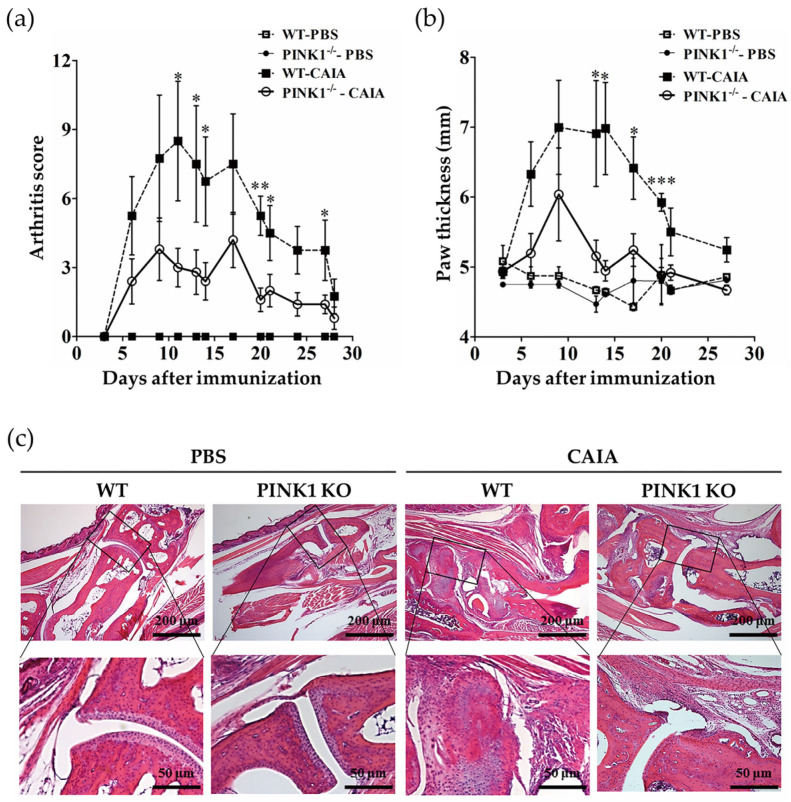
*Pink1* deficiency alleviates collagen antibody-induced arthritis in mice. Three days, 14 days, and 21 days after injection with collagen antibody cocktail, mice received intraperitoneal (i.p) injections of 50 μg lipopolysaccharide. Arthritis incidence and severity were assessed by clinical scoring (**a**) and by measuring paw thickness (**b**) from Days 3–28. Disease severity was graded on a scale of 0–4 as described in the materials and methods. Footpad thickness was measured three times per week. (**c**) Paws were removed from each group of mice after euthanasia, fixed for seven days in 4% paraformaldehyde, decalcified for 21 days in decalcification solution, dehydrated, and embedded in paraffin blocks. Serial sections (10 μm) were cut and stained with hematoxylin and eosin (H&E). Data are expressed as means ± SEM (*n* = 4 mice/group). * *p* < 0.05; ** *p* < 0.01; *** *p* < 0.001.

According to the above findings, we hypothesized that ablating *PINK1* could alleviate collagen antibody-induced arthritis (CAIA) in vivo. The CAIA mouse model is a well-established model of autoimmune arthritis, in which four monoclonal antibodies against collagen and LPS are used to induce arthritis in mice during short periods. CAIA was induced in *Pink1*^−/−^ mice and wild-type mice to determine the effect of PINK1 on CAIA. Multiple experimental repetitions demonstrated that the onset of CAIA was abrogated in *Pink1*^−/−^ mice (Figure 4a,b). We further assessed histopathology in wild-type and *Pink1*^−/−^ CAIA mice. RA histopathology was not present in *Pink1*^−/−^ CAIA mice, while marked infiltration of inflammatory cells and clear pannus formation was present in wild-type CAIA mice (Figure 4c). These results suggest that decreased mitophagy could compromise the function of synovial fibroblasts, which may cause bone infiltration and erosion.

## 3. Discussion

To our knowledge, this is the first report to identify a role for PINK1, a serine/threonine kinase critical for mitophagy, in RA. We demonstrated that PINK1 was more highly expressed in RA synovial membranes than in OA synovial membranes. We also demonstrated that in RASFs treated with TNF-α, PINK1 accumulated on mitochondria to initiate mitophagy. Despite mitochondrial damage in the inflammatory environment, mitophagy maintained homeostasis in synovial fibroblasts, allowing the cells to infiltrate into the joint region and induce arthritis. TNF-α actively induced ROS production and mitochondrial depolarization, which would be expected to trigger PINK1 accumulation on the mitochondrial outer membrane. Knockdown of *PINK1* prevented RASF migration and invasion. Moreover, genetic deletion of *Pink1* modestly ameliorated CAIA. Although recent studies have demonstrated that irregulating PINK1 expression can improve Parkinson’s disease and pulmonary fibrosis, the role of mitophagy in RA had not previously been investigated.

Although loss-of-function PINK1 mutations are a major contributing factor to familial Parkinson’s disease [26], increased PINK1 protein aggregation appears to exacerbate rheumatoid arthritis, contributing to joint destruction. We demonstrated that in RASFs, which contribute to joint breakdown in rheumatoid arthritis, PINK1 knockdown decreased cell invasion and migration, including under TNF-α stimulation. Furthermore, we induced CAIA in *Pink1*^−/−^ mice, revealing that arthritis progressed more slowly in Pink^−/−^ mice relative to wild-type CAIA mice. This suggested that Pink1 deficiency induces the inhibition of mitophagy and accumulation of damaged mitochondria in synovial fibroblasts. The accumulation of dysfunctional mitochondrial in RASFs reduces migration and invasion capacity by increasing oxidative stress.

**Figure 5:**
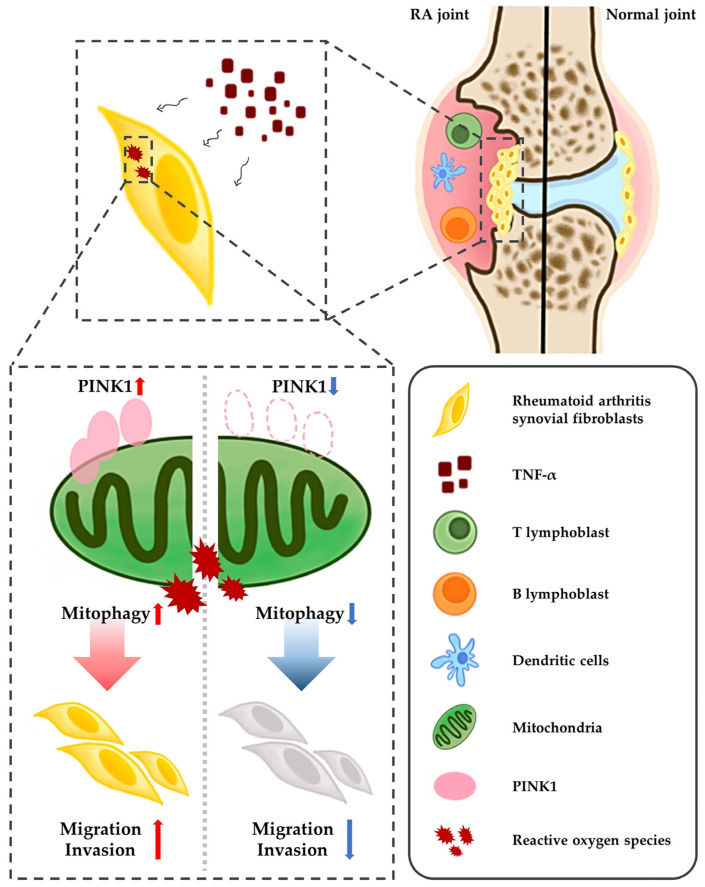
The role of *PINK1*, mitophagy and TNF-α crosstalk in pathogenesis of RA. TNF-α-mediated RASFs proliferation, through the activation of mitophagy, destroys the synovial membrane and cartilage of CAIA mouse model. *PINK1*-deficient RASFs accumulate damaged mitochondria as mitophagy is inhibited, preventing migration and invasion.

In conclusion, the present study demonstrated that PINK1 expression was increased in the synovial membranes of RA patients, and that ablation of Pink1 alleviated arthritis in the murine CAIA model. Furthermore, in synovial fibroblasts, siRNA knockdown of PINK1 decreased invasion potential. Synovial fibroblasts penetrating the joints induce synovitis, so decreased migratory ability of fibroblast could potentially alleviate joint degeneration (Figure 5). Taken together, these findings suggest that inhibiting mitophagy by decreasing *PINK1* gene expression and/or protein stabilization in RASFs is a new putative therapeutic modality for rheumatoid arthritis.

## 4. Materials and Methods

### 4.1. Cell Lines and Reagents

The patient cohort, from which the cell lines established had a median age of 69 years (range 58–80 years). The RASF (passage 5–8) and OASF (passage 3–7) cell lines were obtained from the Catholic University of Korea, Seoul ST. Mary’s Hospital (Seoul, Korea). The cell lines were cultured in Dulbecco’s Modified Eagle Medium (DMEM; Hyclone, GE Healthcare Life Sciences, Logan, UT, USA) containing 10% fetal bovine serum (FBS; Corning, Glendale, NY, USA), and 1 × antibiotic-antimycotic solution (Gibco, Carlsbad, CA, USA) at 37 °C in 5% CO_2_.

### 4.2. Approvals of Animal Experiments

The protocols for the use of animals in these studies were approved by the Institutional Animal Care and Use Committee (IACUC) of CHA University (Project No. 150081) and all experiments were conducted in accordance with the approved protocols.

### 4.3. Mice

The study used female C57BL/6 mice (6–8 weeks-of-age, 14–16 g). Wild type mice were purchased from Orient Bio Inc. (Gyeonggi, Korea). All mice were housed under a 12 h/12 h light/dark cycle in a temperature- and humidity-controlled room. *Pink1*^−/−^ mice were a gift from Prof. Eunhye Joe of Ajou University. *Pink1*^−/−^ mice were generated by replacing a 5.6-kb genomic region of the *Pink1* locus, including exons 4–7 and the coding portion of exon 8, with the PGK-neo-polyA selection cassette flanked by FRT sequences [27].

### 4.4. Western Blotting

Proteins were extracted from RASFs and OASFs using PRO-PREP^TM^ protein extraction buffer (iNtRON Biotechnology Inc., Gyeonggi, Korea). Total protein concentration was measured by Bradford assay using BSA as a standard (Thermo fisher scientific, Waltham, MA, USA). Proteins were separated via 10% or 12% SDS-PAGE and transferred to PVDF membranes (Biorad, Hercules, CA, USA). Membranes were pre-incubated with 5% skim milk in PBS-T (0.1% Tween 20 in PBS) and subsequently incubated with primary antibodies (anti-human GAPDH; Cell signaling, Danvers, MA, USA, anti-human PINK1; Novus bio, Littleton, CO, USA; LC3; Sigma-Aldrich, Burlington, MA, USA, all diluted 1:1000) overnight at 4 °C and incubated with HRP-anti-mouse IgG or HRP-anti-rabbit IgG (1:5000; Cell signaling) for 2 h at room temperature. Membranes were exposed to ECL solution (Thermo fisher scientific, Waltham, MA, USA), and signals were detected using an LAS-4000 luminescent image analyzer (FujiFilm, Tokyo, Japan). Band intensities were quantified using Multi Gauge software V3.0 (Fuji film).

### 4.5. Immunofluorescence

Synovial membrane tissue sections were post-fixed overnight in 4% paraformaldehyde (PFA) at 4 °C and cryoprotected in 30% sucrose for 48 h. Tissue sections were cut at 30 μm using a cryostat. RASFs were cultured on four-well chamber slides (Nalge Nunc international, Rochester, NY, USA) and incubated with 10 ng/mL TNF-α and 10 μM CCCP, commonly used inducer of mitophagy. After 24 h of incubation, RASFs were washed with HBSS (LONZA), stained with 5 nM mitotrakcer deep red (Thermofisher Science, Waltham, MA, USA) for 15 min, and stained with 5 μM CM-DCFDA to detect ROS (Thermofisher science) for 30 min. Samples were fixed with 2% PFA and permeabilized with triton X-100 (Amresco, Solon, OH, USA) and incubated for 1 h in a blocking solution (0.3% Triton-X, 5% normal goat serum in 0.1 M PBS; Cell signaling) and subsequently immunostained with primary antibodies (anti-human PINK1, 1:100;) in antibody diluent (Agilent, Santa Clara, CA, USA) at 4 °C overnight. Slides were then incubated with corresponding fluorescent secondary antibodies for 2 h. A series of fluorescent images were obtained with a Zeiss LSM 510 META system (Carl Zeiss) equipped with a multiargon laser (458, 488, and 514.5 nm) and a He-Ne laser (543 nm). Thirty-millimeter Z stack images in 5-mm steps were processed for further analysis using Zen software (Carl Zeiss).

### 4.6. Measurement of Mitochondria Membrane Potential (ΔΨm)

To determine mitochondrial membrane potential, cells were detached from plates with 0.05% Trypsin-EDTA (Gibco), washed with 1% FBS/DMEM, and incubated with 1 × JC-10 (Mitochondrial membrane potential assay kit, Abcam, Cambridge, UK) for 30 min at 37 °C. The stained cells were analyzed using a BD FACS caliber (Becton, Dickinson and Company, Franklin, NJ, USA). The fluorescence intensities for both J-aggregates and monomeric forms of JC-10 were measured with a flow cytometer using the FL1 and FL2 channels.

### 4.7. Migration and Invasion Assay

Migration was assessed using a Transwell assay with a Transwell^®^ permeable support (Corning, Corning, NY, USA). Briefly, the inner chambers of the transwells containing polycarbonate membrane inserts were seeded with 6 × 10^4^ RASFs transfected with Pink1 or control siRNA. Media containing 10% FBS was added to the lower well of the migration plate. TNF-α was added to the upper well, which contained cells that were activated with TNF-α for 24 h. Migrated cells were stained with crystal violet and extracted with an extraction solution (0.1 M NaH_2_PO_4_: ethanol = 1:1 mixture). The optical density of the extracted solution was measured at 560 nm with a Microplate Reader (Biorad, Hercules, CA, USA). For the invasion assay, an invasion chamber with BD Matrigel Matrix (Corning) was used. This basement membrane layer served as a barrier to discriminate invasive from non-invasive cells. The invasion assay was performed simultaneously using an identical protocol to that of the migration assay.

### 4.8. CAIA Induction

Female C57BL/6 mice were injected intraperitoneally with 500 mg Arthrogen-CIA^®^ 5-Clone Cocktail Kit (Chondrex, Redmond, WA, USA). As WT-PBS controls, mice received equal volumes of PBS vehicle. On days 3, 14, and 21, 50 μg lipopolysaccharide (Chondrex) was injected intraperitoneally into all mice.

### 4.9. Histopathological Examination

None of the control mice developed arthritis. Arthritis score and foot thickness (all four paws) were observed three times weekly at intervals of one or more days until Day 30 post-primary immunization using a triple-blind test. The severity of arthritis was expressed as the mean arthritis index, graded on a scale of 0–4 (0, no arthritis; 1, light edema at the point of one finger; 2, edema at several points on a finger or in the joints of the wrist or ankle; 3, pervading edema involving the entire paw; 4, maximal pervading edema involving the entire paw and deformation of the joints (ankylosis) with impaired function). The maximum total arthritis score that each mouse could receive was 16. On Day 30, all mice were sacrificed, and paws were collected. Paws from each group were fixed in 4% PFA, decalcified in decalcifying solution (Sigma-Aldrich, Burlington, MA, USA) diluted 1:10 with deionized water and embedded in paraffin. Serial sections (10 μm) were prepared and stained with hematoxylin and eosin (H&E).

### 4.10. Statistical Analyses

Statistical analyses were performed using GraphPad software (GraphPad Prism v5.0; GraphPad Software, San Diego, CA, USA). The Shapiro–Wilk test was used to check for normality requirements. Data that fulfilled the normality were analyzed using a Student’s t-test (two variables) or one-way ANOVA (≥3 independent groups) followed by the Newman–Keuls test. Non-normally distributed data were examined using the Kruskal–Wallis test (≥3 independent groups) with Bonferroni correction. Results were expressed as means ± SEM. A *p*-value < 0.05 was considered statistically significant. * *p* < 0.05, ** *p* < 0.01, *** *p* < 0.001.

## Abbreviations

RA	Rheumatoid arthritis
RASFs	Rheumatoid arthritis synovial fibroblasts
PINK1	PTEN-induced putative kinase1
TNF-α	Tumor necrosis factor-alpha
CCCP	Carbonyl cyanide 3-chlorophenylhydrazone
NAC	N-acetylcysteine
CAIA	Collagen antibody-induced arthritis
ROS	Reactive oxygen species
TNF-α	Tumor necrosis factor-alpha
